# Dimerization of 30Kc19 protein in the presence of amphiphilic moiety and importance of Cys-57 during cell penetration

**DOI:** 10.1002/biot.201400253

**Published:** 2014-09-10

**Authors:** Hee Ho Park, Youngsoo Sohn, Ji Woo Yeo, Ju Hyun Park, Hong Jai Lee, Jina Ryu, Won Jong Rhee, Tai Hyun Park

**Affiliations:** 1 The School of Chemical and Biological Engineering, Seoul National University, SeoulRepublic of Korea; 2 Interdisciplinary Program for Bioengineering, Seoul National UniversitySeoul, Republic of Korea; 3 Division of Bioengineering, Incheon National UniversityIncheon, Republic of Korea; 4 Advanced Institutes of Convergence TechnologySuwon, Republic of Korea

**Keywords:** Dimerization, 30Kc19 protein, Penetration, Phospholipid, Sodium dodecyl sulfate

## Abstract

Recently, the recombinant 30Kc19 protein, originating from silkworm hemolymph of *Bombyx mori* has attracted attention due to its cell-penetrating property and potential application as a protein delivery system. However, this observation of penetration across cell membrane has raised questions concerning the interaction of the protein-lipid bilayer. Here, we report a dimerization propensity of the 30Kc19 protein in the presence of amphiphilic moieties; sodium dodecyl sulfate (SDS) or phospholipid. Native PAGE showed that the 30Kc19 monomer formed a dimer when SDS or phospholipid was present. In the glutathione-*S*-transferase (GST) pull-down assay, supplementation of the 30Kc19 protein to mammalian cell culture medium showed dimerization and penetration; due to phospholipids at the cell membrane, the main components of the lipid bilayer. Mutagenesis was performed, and penetration was observed by 30Kc19 C76A and not 30Kc19 C57A, which meant that the presence of cysteine at position 57 (Cys-57) is involved in dimerization of the 30Kc19 at the cell membrane during penetration. We anticipate application of the native 30Kc19 protein with high cell-penetrating efficiency for delivery of cargos to various cell types. The intracellular cargo delivery using the 30Kc19 protein is a non-virus derived (e.g. TAT) delivery method, which can open up new approaches for the delivery of therapeutics in bioindustries, such as pharma- and cosmeceuticals.

## 1 Introduction

The 30Kc19 protein, a member of the 30K protein family, is a similar structured protein found in silkworm hemolymph, *Bombyx mori* [1]. It is the most abundant among 30K proteins (30Kc6, 30Kc12, 30Kc19, 30Kc21, and 30Kc23) in hemolymph with molecular weights of about 30 kDa [2]. These “30K proteins” are synthesized in fat body cells and accumulate in the hemolymph during the fifth instar larval to early pupal stages [3, 4]. They are then transferred from the hemolymph to fat body cells during metamorphosis from larva to pupa and are deposited there until use [5, 6].

The biological functions of the 30K proteins in silkworms have not been fully determined, although several studies have recently examined their functional properties [6, 7]. Previously, we have demonstrated that silkworm hemolymph and 30K proteins exhibit an anti-apoptotic effect in various cells by adding them to culture medium or by gene expression [8–20]. Other than the anti-apoptotic effect, 30K proteins also enhance production of recombinant erythropoietin, interferon-β, and monoclonal antibodies; increase glycosylation, cell growth, and viability in various cells; and have an enzyme-stabilizing effect [21–28]. A previous study showed the presence of the 30Kc19 protein inside cultured cells when supplemented to the culture medium [29]. Therefore, the 30Kc19 protein is a very unique multifunctional protein that can be applied for the delivery of therapeutic proteins including enzymes, as it can penetrate cell membranes and stabilize cargo proteins. It is necessary to understand the molecular mechanism of cell penetration for the practical use of the 30Kc19 protein. However, the exact mechanism of penetration to animal cells has not been fully determined.

Herein, we report a dimerization propensity of the 30Kc19 protein in the presence of either sodium dodecyl sulfate (SDS) or phospholipids. We investigated how the cell-penetrating 30Kc19 protein is related with phospholipids, the main cell membrane components, and elucidated the mechanism of entry of the 30Kc19 protein into animal cells for use in protein delivery system. The 30Kc19 protein is a non-virus derived (e.g. TAT) cell-penetrating protein (CPP), thus may open up new approaches for the delivery of therapeutics in bioindustries, such as pharma- and cosmeceuticals.

## 2 Materials and methods

### 2.1 Construction of expression vectors

Total RNA was isolated from *B. mori* silkworm at the fifth-instar larval stage using RNeasy (Qiagen, Valencia, CA, USA), and 30Kc19 cDNA was obtained by RT-PCR. The 30Kc19 gene was amplified using PCR, and the DNA fragment was inserted into the pET-23a expression vector (Novagen, Madison, WI, USA) with a T7 tag at the N-terminus and a 6-His tag at the C-terminus. The glutathione-*S*-transferase (GST)-30Kc19 ORFs were cloned from the pGEX-4T-1 vector (GE Healthcare, Uppsala, Sweden) into the N-terminal of 30Kc19 in the pET-23a vector. The GST-30Kc19 fusion protein contained two amino acids (Glu and Phe) derived from the *Eco*RI sequence (GAATTC) between GST and 30Kc19. Point mutation of pET-23a/*30Kc19* was requested and performed by Enzynomics and pET-23a/*30Kc19* *C57A* and pET-23a/*30Kc19 C76A* were constructed. For GFP-30Kc19, ORFs of GFP were cloned from pCMV-AC-GFP vector (Origene, Rockville, MD, USA) to N-terminal of 30Kc19 in pET-23a vector. The GFP-30Kc19 contained two amino acids (Glu, Phe) derived from the *Eco*RI sequence (GAATTC) between GFP and 30Kc19.

### 2.2 Protein expression and purification

The constructed vector was transformed into *Escherichia coli* BL21 (DE3, Novagen) and cells were grown in LB-ampicillin medium at 37°C. Isopropyl 1-thio-β-d-galactopyranoside (IPTG, 1 mM) was used for induction, and all proteins were further incubated at 37°C for the production of protein, except for GFP-30Kc19, for which 30°C was selected as the induction temperature. After centrifugation, the cells were harvested and disrupted by sonication. Following cell lysis, all recombinant proteins except GST-fusion protein were purified from the supernatant using a HisTrap HP column (GE Healthcare), dialyzed against 20 mM tris–HCl buffer (pH 8.0) using a HiTrap desalting column (GE Healthcare) with purity >90% (data not shown), and stored at –70°C until use. For the GST-fusion protein, the purified protein was dialyzed against PBS (pH 7.4) and 300 mM NaCl and stored at –70°C until use. The quantitative analysis of proteins was performed using a Micro BCA kit (Thermo Fisher Scientific, Inc., Rockford, IL, USA).

### 2.3 Reducing SDS–PAGE, non-reducing SDS–PAGE, and native PAGE

All reducing SDS–PAGE, non-reducing SDS–PAGE, and native PAGE was conducted using 12% polyacrylamide gels. For the reducing condition, samples were mixed with reducing sample buffer containing SDS and β-mercaptoethanol (BME) (pH 6.8), and for non-reducing condition, samples were mixed with non-reducing sample buffer without BME. Fifteen minutes pre-incubation of 30Kc19 proteins with SDS, detergents, and materials were performed prior to loading. The reducing condition samples with the reducing buffer were denatured by boiling. For the native condition, samples were mixed with native sample buffer without any denaturing reagent. After electrophoresis, each sample was separated according to size (reducing or non-reducing) or pattern (native). The polyacrylamide gel was immersed in Coomassie blue staining solution and then immersed in destaining solution for analysis.

A 42 and 67 kDa sized ovalbumin and BSA (Sigma, St. Louis, MO, USA) were used as standards for the molecular weight assay of the recombinant 30Kc19 protein. SDS (Sigma) was dissolved and diluted with deionized water (DW) according to the appropriate concentration for the molecular weight assay. Cetyl trimethylammonium bromide (CTAB), Triton X-100, and 3-[(3-cholamidopropyl)dimethylammonio]-1-propanesulfonate (CHAPS) detergents (all from Sigma) were dissolved in DW and stored in frozen aliquots until use. Dextran sulfate sodium salt (Fluka) was dissolved in DW and used in the experiment. l-α-Phosphatidyl choline (Sigma) was used as the phospholipid. It was dissolved in chloroform and stored at –20°C until use. Prior to the experiment, the chloroform was removed with nitrogen gas and diluted with DW. Five controlled cycles of freeze–thawing were carried out to form unilamellar vesicles. Unless otherwise indicated, 1 mg/mL of proteins were used in the experiment.

### 2.4 Size exclusion chromatography (SEC)

High performance liquid chromatography (HPLC) (Waters, Milford, MA, USA) was used for size exclusion of 30Kc19. Prior to sample loading, the column (Tosoh, TSK-GEL G3000 SWXL 7.8 nm × 300 nm column) was pre-equilibrated with sodium phosphate buffer (50 mM sodium phosphate, 150 mM NaCl, pH 7.0). Then, 50 μL of sample was injected with an automated sample injection system. The flow rate was 1 mL/min, and the detection wavelength was 280 nm.

### 2.5 Immunoblot analysis

HEK-293 cells were maintained in a humidified atmosphere of 5% CO_2_ at 37°C in DMEM supplemented with 10% w/v fetal bovine serum (FBS; Gibco, Grand Island, NY, USA) and 1% v/v penicillin streptomycin (PS; Gibco). Protein was added to the culture medium and incubated for 6 h at 37°C in a humidified atmosphere of 5% CO_2_. After the incubation, cells were treated with trypsin–EDTA (Sigma) then washed three times with PBS. Cells were treated with trypsin–EDTA to distinguish between intracellular and membrane-bound proteins (Sigma). The collected cells were washed three times in PBS. Cell extracts were collected with RIPA buffer (50 mM tris–HCl (pH 7.4), 150 mM NaCl, 1% Triton X-100, 0.1% SDS, protease inhibitor cocktail) at 4°C for 1 h followed by centrifugation. Each cell extract containing an equal amount of protein was resolved by PAGE and examined by immunoblot analysis. Anti-30Kc19 rabbit antibody was prepared using the following procedure. 30Kc19 was first purified from silkworm hemolymph using a two-step chromatography purification method (size exclusion and ion exchange). Anti-30Kc19 polyclonal antibody was produced by immunizing a rabbit with the purified 30Kc19 protein, which was subsequently purified by Protein G chromatography (AbFrontier, Seoul, Korea). 30Kc19 was detected using this anti-30Kc19 antibody and an HRP-conjugated anti-rabbit antibody (Invitrogen, Carlsbad, CA, USA).

### 2.6 GST pull-down assay

Purified GST-tagged proteins were prebound to resin by incubating the proteins with GST-bind resin for 2 h at 4°C in PBS (pH 7.4), 300 mM NaCl, and a protease inhibitor mixture. The prebound resin was washed three times with the same buffer solution. Then, samples were analyzed by immunoblotting with the anti-30Kc19 rabbit antibody, followed by an HRP-conjugated anti-rabbit antibody.

### 2.7 Fluorescence microscopy

For immunocytochemistry, cell penetration of the protein was visualized using confocal microscopy. HeLa cells were seeded on 8-well chamber slides (Nunc Lab-Tek, Rochester, NY, USA) and incubated overnight. Protein was added to the culture medium and incubated for 6 h at 37°C in a humidified atmosphere of 5% CO_2_. After the incubation, the cells were treated with trypsin–EDTA (Sigma) and washed three times with PBS, fixed in 4% paraformaldehyde for 20 min, and incubated for 10 min with 0.25% Triton X-100 in PBS for permeabilization. The fixed cells were blocked with 3% BSA in 0.1% PBS-T for 1 h and then incubated with anti-30Kc19 rabbit antibody (Ab Frontier) and either rhodopsin-conjugated anti-rabbit antibody (Jackson ImmunoResearch, West Grove, PA, USA) or Alexa Fluor 488-conjugated anti-rabbit antibody (Invitrogen) were used for the secondary antibody. Nuclei of cells were stained with Hoechst 33342 for 10 min. A confocal laser microscope (EZ-C1, Nikon, Tokyo, Japan) was used to observe intracellular fluorescence, and images were taken using the manufacturer's software (Nikon).

For live cell analysis, protein was added to the culture medium and incubated for 6 h at 37°C in a humidified atmosphere of 5% CO_2_. After the incubation, the cells were treated with trypsin–EDTA (Sigma) and washed three times with PBS, nuclei of cells were then stained with Hoechst 33342, washed and then live cell intracellular fluorescence and images were taken.

### 2.8 Inhibitors of endocytosis

When cell-penetrating efficiency was performed in the presence of cytochalasin B (25 μM), sucrose (100 nM), or nystatin (25 μg/mL) (all purchased from Sigma–Aldrich), cells were preincubated with these inhibitors of endocytosis for 1 h prior to supplementation of 30Kc19 protein to culture medium. Incubation was performed for 6 h, after which cells were extensively washed analyzed by immunocytochemistry method as mentioned previously using spectrofluorometer in order to determine the intracellular penetration efficiency.

## 3 Results

### 3.1 Cell-penetration of 30Kc19 protein

**Figure 1:**
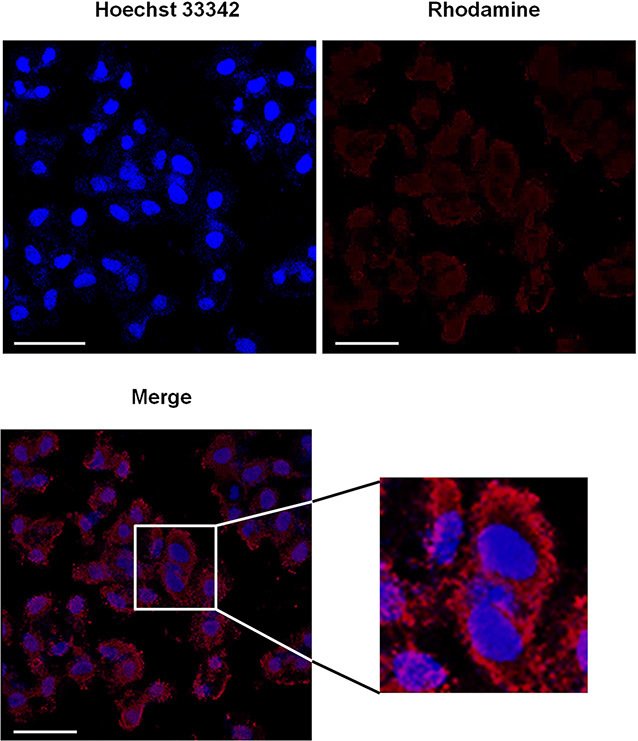
Cell penetration property of the 30Kc19 protein. HeLa cells were supplemented with the 30Kc19 protein in culture medium for 6 h. The cell penetration ability of the 30Kc19 protein was analyzed by immunocytochemistry, which showed internalization of 30Kc19 protein. The internalized protein was visualized by rhodopsin-conjugated anti-rabbit antibody (red), and nuclei were visualized with Hoechst 33342 (blue). Supplementing the cell culture medium with the protein was conducted in a quantity of 0.2 mg/mL. Scale bar, 50 μm.

30Kc19 protein, comprised of 239 amino acids in total, has all-helix in N-terminal domain and all-β sheet in C-terminal domain [30, 31]. This 30Kc19 protein has recently shown a cell-penetrating property in various types of cells when supplemented to culture medium and was found to be the first CPP in insect hemolymph that exhibited a cell-penetration property both in vitro and in vivo [32]. In this study, we investigated the cell-penetrating property and the intracellular penetration mechanism of the 30Kc19 protein. First, 30Kc19 protein was added to culture medium and immunocytochemical analysis was conducted using anti-30Kc19 rabbit antibody and rhodamine-conjugated anti-rabbit antibody. Detection of red color demonstrated internalization of the 30Kc19 protein (Fig. 1). The results showed that 30Kc19 protein was localized in the cytoplasm of the cell.

### 3.2 Dimerization of the 30Kc19 protein is promoted by SDS

**Figure 2:**
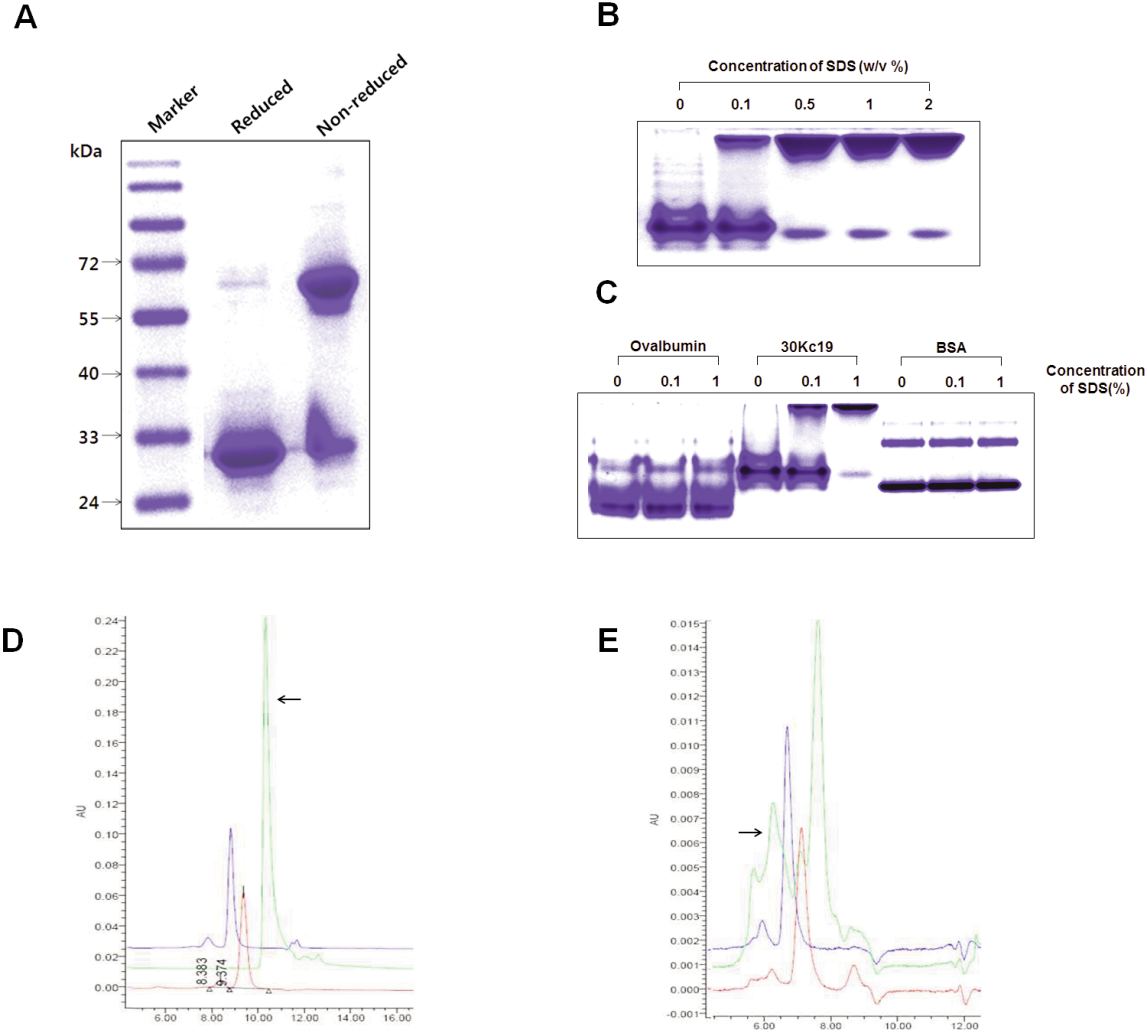
Monomer and dimer forms of 30Kc19. (A) SDS–PAGE result of the 30Kc19 protein under reducing and non-reducing conditions. (B) Native PAGE result of the 30Kc19 protein pre-treated with different SDS concentrations. (C) Native PAGE result of the 30Kc19, ovalbumin, and BSA proteins, each pre-treated with different SDS concentrations. (D) SEC result of 30Kc19, ovalbumin, and BSA proteins without SDS pre-treatment and with 1% w/v SDS pre-treatment. Concentration of 1 mg/mL was used for all proteins; 30Kc19, ovalbumin, and BSA.

Recently, we have seen a dimerization propensity of the 30Kc19 protein during characterization assay using PAGE, and we believe this dimerization is relevant for cell penetration. The 30Kc19 protein was run on PAGE under reducing and non-reducing conditions and observed the dimerization propensity. Under the reducing condition, a monomer and a faint dimer band were seen (Fig. 2A). In contrast, not only a 30 kDa sized monomer protein was detected under a non-reducing condition but also a clear 60 kDa dimer sized protein was detected.

These results demonstrated that the properties of the 30Kc19 protein are similar to other peptides under reducing and non-reducing conditions, dimer was considered to be due to the non-reducing environment in the presence of SDS [33, 34]. To confirm whether this was the case, 30Kc19 was pre-treated with different concentrations of SDS for 10 min and was then loaded on native PAGE. A monomer band was detected when no SDS was mixed with the protein. However, both a monomer and dimer were detected when the SDS concentration was increased to 0.1% (Fig. 2B). A shift from the monomer to the dimer was detected in 0.1% SDS, and almost all monomers shifted to the dimer at 0.5% SDS. Therefore, 30Kc19 originally existed as a monomer and dimerized in the presence of SDS. Additional experiments were carried out to verify this for other well-known standard proteins such as ovalbumin and BSA. Ovalbumin and BSA were loaded on native PAGE but showed no significant difference in pattern as the concentration of SDS increased when compared with that of 30Kc19 (Fig. 2C). We carried out size exclusion chromatography (SEC) using HPLC to confirm that pre-treatment with SDS actually resulted in an increased size of the 30Kc19 protein. The experiment was carried out without prior SDS treatment and no SDS in the buffer. The sequential order of the proteins that passed through the column was in the order: BSA (66 kDa), ovalbumin (42 kDa), and 30Kc19 (30 kDa), with times of 8.75, 9.4, and 10.5 s, respectively (Fig. 2D). Adding SDS to the column shortened the overall retention times of the peaks, which is generally observed in SEC [35]. When the 30Kc19 was pre-treated with 1% SDS and when SDS was added to the running buffer at a final concentration of 1%, comparative differences in the peak patterns were seen. The pre-treatment shortened the retention time of the 30Kc19 protein, which was detected earlier than ovalbumin and BSA (Fig. 2E). This result shows that there was an increase in the size of 30Kc19, indicating dimerization and multimerization of the protein. Taken together, we concluded that the 30Kc19 protein exists as a monomer and that SDS causes multimerization of 30Kc19.

### 3.3 Dimerization of the 30Kc19 protein is promoted by phospholipid

**Figure 3:**
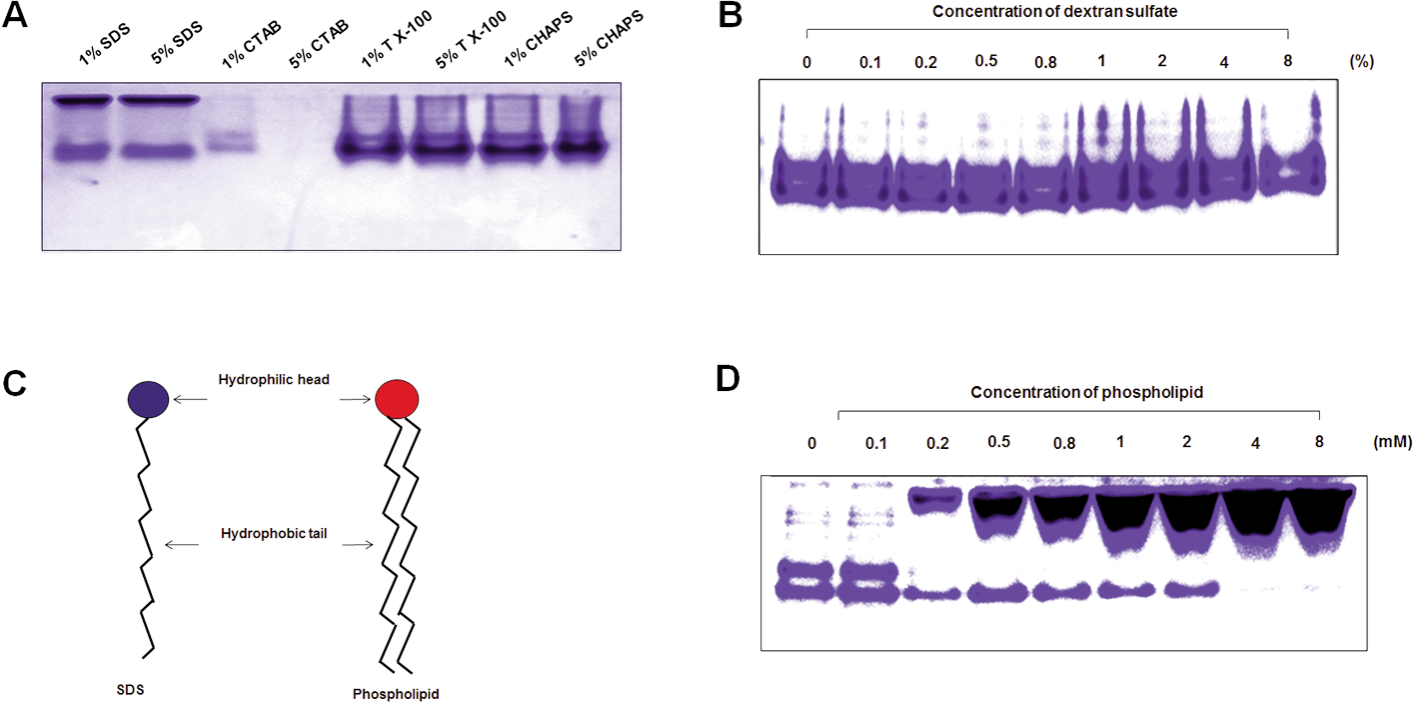
Monomer and dimer forms of 30Kc19. (A) Native PAGE result of the 30Kc19 protein pre-treated with different classes of anionic (SDS), cationic (CTAB), non-ionic (T X-100), and zwitterionic (CHAPS) surfactant detergents. (B) Native PAGE result of the 30Kc19 protein pre-treated with negative material, dextran sulfate. (C) Schematic diagram showing similarity of SDS and phospholipids. (D) Native PAGE result of the 30Kc19 protein pre-treated with different phospholipid concentrations.

Dimerization/multimerization propensity was seen for 30Kc19 when SDS was mixed with the protein. It was unclear whether this occurred because SDS is a surfactant. SDS is an anionic (negative) detergent; hence, cationic, non-ionic, and zwitterionic detergents were selected and tested for dimerization [36]. When the cationic surfactant CTAB was mixed with the 30Kc19 protein, no dimerization was observed (Fig. 3A). In fact, because CTAB is a positively charged detergent, the 30Kc19-CTAB mixture did not progress on PAGE. Triton X-100 and CHAPS, which are non-ionic and zwitterionic surfactants, respectively, resulted in no dimerization of 30Kc19, indicating that dimerization of 30Kc19 is not just dependent on the surfactant nature of SDS and that it could be due to the anionic property of SDS. We hypothesized that dimerization may have been caused by the negative SDS charge. Hence a well-known polyanionic material, dextran sulfate, was used in various concentrations to determine the reason for the change in the 30Kc19 pattern [32]. However, when dextran sulfate was added, no difference in the 30Kc19 pattern was seen (Fig. 3B). We found l-α-phosphatidylcholine, a phospholipid, which is similar in structure and properties to SDS, has an amphiphilic moiety and is a component of the cell membrane with similar structural properties to SDS (Fig. 3C). When a low concentration of phospholipid (0.1 mM) was mixed with the 30Kc19 protein, no tendency for a pattern shift was detected. However, as the concentration increased, a significant difference in the 30Kc19 protein pattern was detected and dimers were seen (Fig. 3D). When the lipid concentration reached 1.5 mM, most of the monomeric 30Kc19 protein was in a dimer form. This result showed that majority of 30Kc19 shifted toward the dimer as the 30Kc19 protein was mixed with increasing concentrations of phospholipid. This observation paralleled with the results shown when the 30Kc19 protein was mixed with SDS, a material with similar properties to phospholipid.

### 3.4 Dimerization of the 30Kc19 protein during penetration to cells through the lipid bilayer membrane

**Figure 4:**
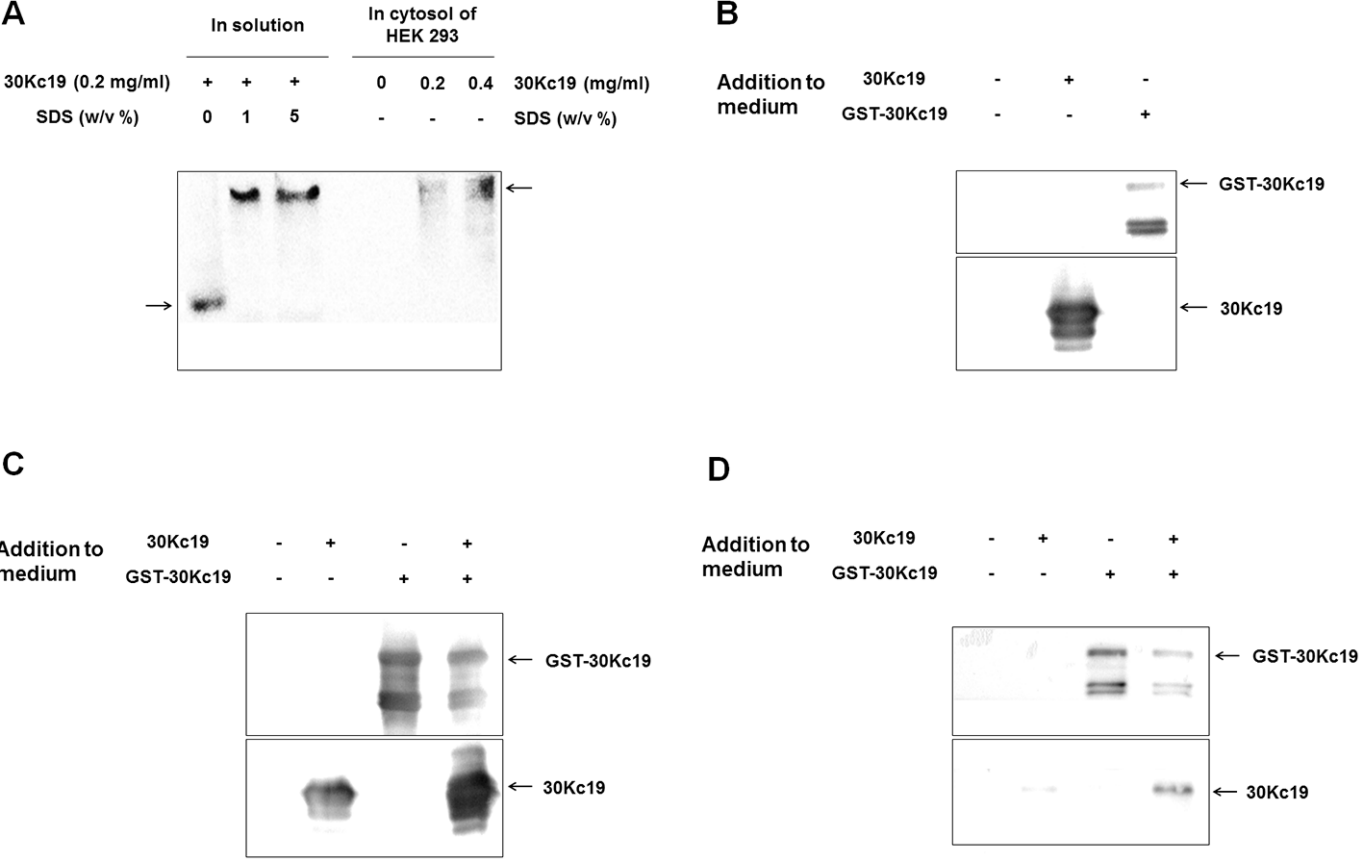
Dimerization of 30Kc19 during cell penetration. (A) Native PAGE immunoblot result of the 30Kc19 protein pre-treated with SDS (left) and cell lysate supplemented with 30Kc19 in culture medium for 6 h (right). (B) Reducing SDS–PAGE result of the 30Kc19 and GST-30Kc19 fusion proteins in solution. (C) Reducing PAGE immunoblot result of the HEK-293 cell lysate, supplemented with the 30Kc19 protein only, the GST-30Kc19 protein only, or the 30Kc19 plus GST-30Kc19 proteins in culture medium for 6 h. (D) HEK-293 cells were supplemented with the 30Kc19 protein only, the GST-30Kc19 protein only, or the 30Kc19 plus the GST-30Kc19 proteins in culture medium for 6 h. Reducing PAGE immunoblot result of the cell lysate GST pull-down assay. Proteins were supplemented in the culture medium in equal molar quantities of 0.2 and 0.4 mg/mL for the 30Kc19 protein and GST-30Kc19 protein, respectively.

Previously, our group identified the cell-penetrating property of the 30Kc19 protein when it is supplemented in culture medium [32]. Phospholipids are a major component of the cell membrane lipid bilayer. We hypothesized the possible relevance of 30Kc19 dimerization with phospholipids and the cell-penetrating property of the 30Kc19 protein. We also elucidated the mechanism of entry of the 30Kc19 protein into animal cells through an interaction with the cell membrane. First, an immunoblot against the 30Kc19 protein incubated with or without SDS is shown as a reference for the pattern analysis (Fig. 4A, left). As expected, the ratio of 30Kc19 dimer to monomer increased by adding SDS to the solution. HEK-293 cells were incubated with 0, 0.2, and 0.4 mg/mL recombinant 30Kc19 protein. The cells were treated with trypsin and were washed multiple times for the removal surface-bound proteins by digestion to distinguish between membrane-bound proteins and intracellular proteins [37, 38]. The cells were then lysed and the lysate was loaded onto native PAGE followed by an immunoblot against the 30Kc19 protein. We observed penetration of the 30Kc19 protein into the cell and detected an increased amount of intracellular 30Kc19 protein as the concentration was raised in the culture medium (Fig. 4A, right). Interestingly, the 30Kc19 protein was found mainly as a dimer in the cell lysate, indicating that the 30Kc19 monomer protein in the medium dimerized during cell penetration or in the cytosol. We further confirmed the dimerized form of the 30Kc19 protein in the cytosol using a GST pull-down assay followed by a reducing PAGE immunoblot. GST-30Kc19 was produced in *E. coli* and loaded on reducing PAGE (Fig. 4B). A 60 kDa sized monomer GST-30Kc19 protein was detected under reducing conditions, and we confirmed that the protein was expressed and purified successfully. Then, the 30Kc19 protein, the GST-30Kc19 protein, or 30Kc19 protein plus GST-30Kc19 protein, respectively, was added to HEK-293 cell culture medium. The cell lysates were loaded onto reducing PAGE, and we confirmed that both the 30Kc19 protein and GST-30Kc19 penetrated the cells (Fig. 4C). When the 30Kc19 protein-treated cell lysate was subjected to the GST pull-down assay, no 30Kc19 protein was detected, as expected, whereas the GST-30Kc19 protein was detected when the GST-30Kc19 protein-treated cell lysate was used. However, when the cell lysate treated with both the 30Kc19 protein and GST-30Kc19 protein was subjected to the GST pull-down assay, the 30Kc19 protein was detected. Based on these results, we conclude that the 30Kc19 protein formed a dimer with 30Kc19 of GST-30Kc19 during penetration and remained a dimer inside the cells (Fig. 4C). Therefore, we suggest that the 30Kc19 protein interacted with phospholipid on the plasma membrane and formed a dimer and this allowed the 30Kc19 protein to penetrate the cells.

### 3.5 Dimerization of the 30Kc19 protein during cell penetration is promoted by Cys-57

**Figure 5:**
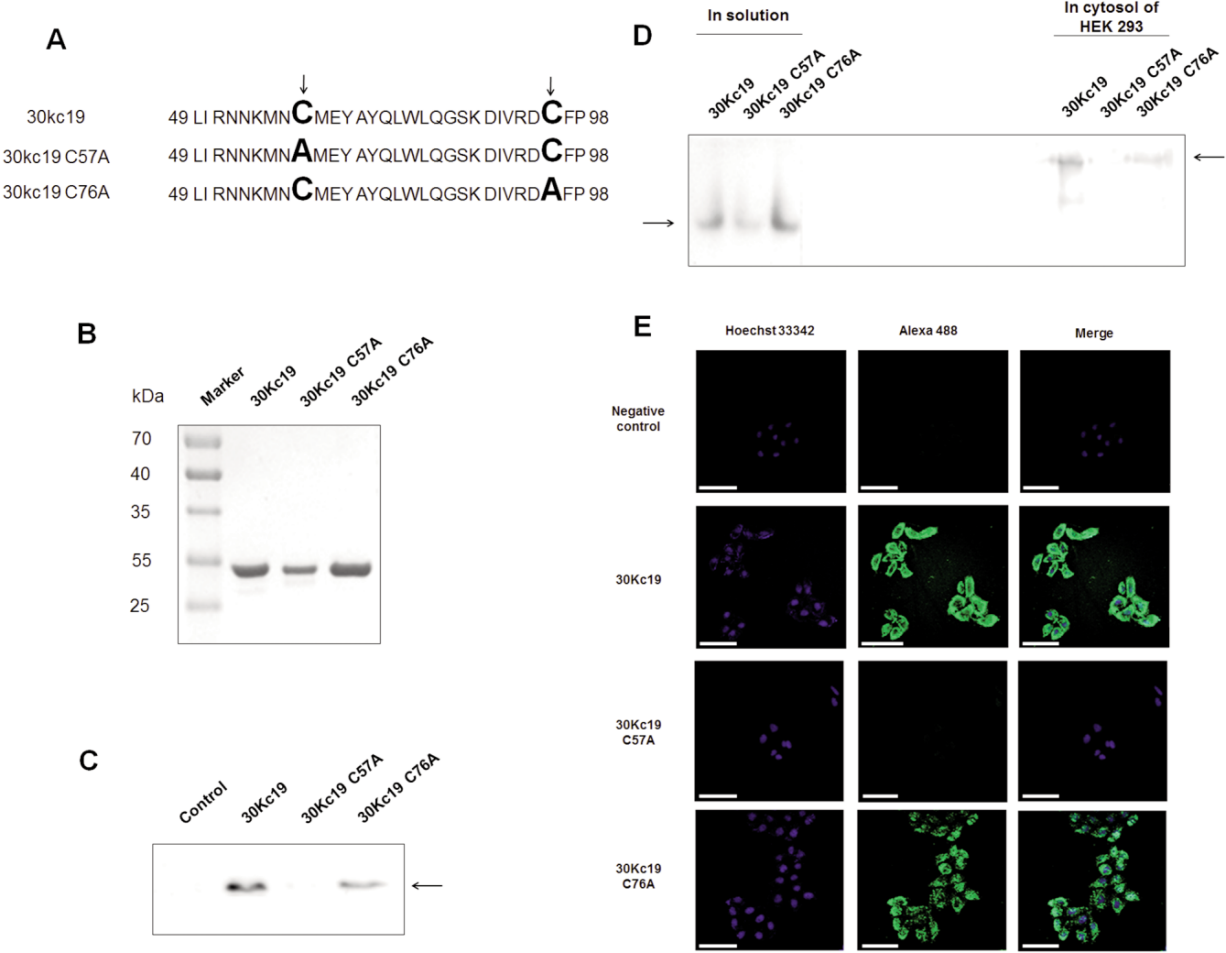
Importance of 30Kc19 Cys-57 for cell penetration. (A) Alignment of 30Kc19 and mutated 30Kc19 with the cysteine and alanine residues highlighted in bold. Arrow points to the cysteine conserved in 30Kc19 (Cys-57 and Cys-76). (B) Reducing SDS–PAGE result of the 30Kc19 protein, 30Kc19 C57A, and 30Kc19 C76A. (C) Reducing PAGE immunoblot result of the HEK-239 cell lysate supplemented with the 30Kc19 protein only, the 30Kc19 C57A protein only, or the 30Kc19 C76A protein in culture medium for 6 h. (D) Native PAGE immunoblot result of the 30Kc19 protein, 30Kc19 C57A, 30Kc19 C76A (left), and the HEK-239 cell lysate supplemented with the 30Kc19 protein only, the 30Kc19 C57A protein only, or the 30Kc19 C76A protein in culture medium for 6 h (right). Monomeric and dimeric species are indicated by arrows. (E) HeLa cells were supplemented with the 30Kc19 protein only, the 30Kc19 C57A protein only, or the 30Kc19 C76A protein only in culture medium for 6 h. The cell penetration ability of the 30Kc19 mutants was analyzed by immunofluorescence, which showed internalization of 30Kc19 and 30Kc19 C76A. The internalized protein was visualized by Alexa Fluor 488 (green), and nuclei were visualized with Hoechst 33342 (blue). Supplementing the cell culture medium with the proteins was conducted in an equal molar quantity of 0.4 mg/mL for all proteins. Scale bar, 50 μm.

We were convinced that the 30Kc19 protein dimerizes during cell penetration but were unsure exactly how the 30Kc19 protein homodimerizes during penetration. A similar result was demonstrated with the peptide hormone resistin in which a disulfide-linked homodimer was converted to a monomer under reducing conditions and conversion of a single cysteine to alanine abolished the dimerization [39, 40]. The 30Kc19 protein has two cysteine residues, Cys-57 and Cys-76 (Fig. 5A). There is high probability that one of these cysteines is involved in dimerization of the 30Kc19 protein. Thus, a point mutation in pET-23a/*30Kc19* was requested and pET-23a/*30Kc19 C57A* and pET-23a/*30Kc19 C76A* were constructed (Enzynomics). Expression and purification of the 30Kc19 C57A and 30Kc19 C76A proteins were performed by the same method as for the 30Kc19 protein, and they were analyzed by reducing SDS–PAGE (Fig. 5B). The result showed that both proteins were expressed successfully. Then, the 30Kc19 protein, the 30Kc19 C57A protein, and the 30Kc19 C76A protein were added in equal amounts (0.4 mg/mL) to culture medium containing HEK-293 cells. The cell lysates were loaded onto reducing PAGE, and we confirmed that the 30Kc19 protein and 30Kc19 C76A penetrated the cells by immunoblot analysis. However, the 30Kc19 C57A protein-treated cell lysate showed no sign of the penetration, indicating that the 30Kc19 protein cannot penetrate cells without Cys-57 (Fig. 5C). We also checked for the dimerization propensity on native PAGE of 30Kc19 C57A and 30Kc19 C76A by immunoblot analysis (Fig. 5D). Similar to the wild-type 30Kc19 protein, 30Kc19 C57A, and 30Kc19 C76A showed no signs of dimerization in the absence of cell penetration (Fig. 4D). However, when each of the protein-treated HEK-293 cell lysates was loaded onto native PAGE for immunoblot analysis, we observed that only the 30Kc19 protein and 30Kc19 C76A penetrated the cells and existed as dimers inside the cells (Fig.5D). Immunocytochemistry of the 30Kc19 proteins was used to confirm penetration of 30Kc19 and 30Kc19 C76A but not 30Kc19 C57A. Note that 30Kc19 C57A was not detected inside the cells, but 30Kc19 C76A managed to penetrate the cells (Fig. 5E). Thus, conversion of this cysteine to alanine at 57 abolished dimerization of 30Kc19, indicating that a single disulfide bond was necessary to connect the two 30Kc19 proteins into a homodimer and that 30Kc19 protein can only penetrate the cell membrane by dimerization. The cysteine residue at 57 is critical for cell penetration, which means it is involved in dimer formation between the 30Kc19 proteins.

### 3.6 Intracellular penetration in the presence of inhibitors of endocytosis

**Figure 6:**
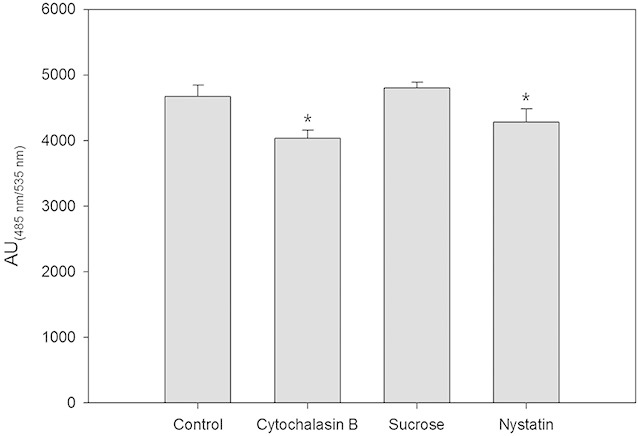
Intracellular penetration in the presence of inhibitors of endocytosis. Cells were pre-incubated for 1 h with different modulators of endocytosis; cytochalasin B (25 μM) for the inhibition of macropinocytosis, sucrose (100 mM) for the inhibition of clathrin-mediated endocytosis, and nystatin (25 μg/mL) for the disruption of caveolar structure and function. 30Kc19 protein was supplemented to the medium. After 6 h of incubation, HeLa cells were fixed with paraformaldehyde, followed by permeabilization with Triton X-100. The intracellular 30Kc19 protein was analyzed by anti-30Kc19 rabbit antibody (primary) and Alexa Fluor 488-conjugated anti-rabbit antibody (secondary). Then fluorescence was measured using spectrofluorometer (ex. 485 nm/em. 535 nm). *p<0.001, compared with the control group (*n* = 4). Error bars represent standard deviation.

Endocytosis is categorized into three pathways such as macropinocytosis, clathrin-mediated endocytosis, and caveolin-mediated endocytosis. Cells were treated with inhibitors of endocytosis; cytochalasin B, sucrose, and nystatin, for the inhibition of macropinocytosis, clathrin-mediated endocytosis, and caveolin-mediated endocytosis, respectively. To minimize the cell stress by inhibitors, we used concentrations that presented minimal toxicity to cells [41]. When cells were given a hyperosmolar condition by sucrose treatment, no markedly difference in the penetration ability of the 30Kc19 protein was observed (Fig. 6). This showed that 30Kc19 protein does not penetrate by clathrin-mediated endocytosis. However, treatment of cytochalasin B or nystatin decreased the cell-penetrating ability of the 30Kc19 protein, which demonstrated that 30Kc19 protein penetrated cells by macropinocytosis and caveolin-mediated endocytosis.

### 3.7 Intracellular delivery of GFP using 30Kc19 protein as a fusion partner

**Figure 7:**
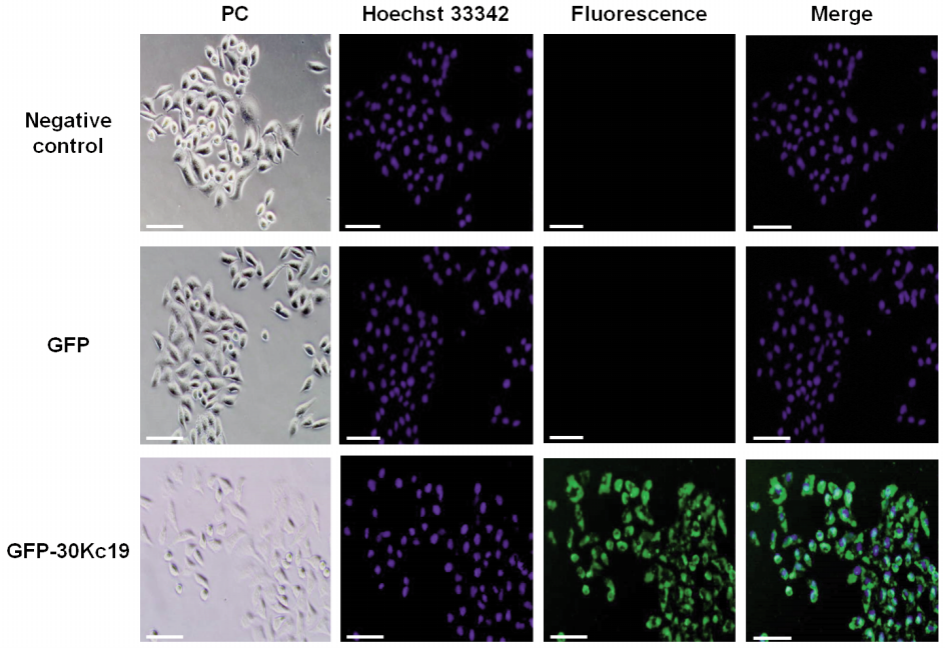
Intracellular cargo delivery using the 30Kc19 protein. HeLa cells were supplemented with the GFP or GFP-30Kc19 fusion protein in culture medium for 6 h. Ability of the 30Kc19 protein to intracellularly deliver GFP fusion protein (GFP-30Kc19) was analyzed by immunofluorescence, which showed internalization of GFP-30Kc19 protein. The internalized protein was visualized by its own signal of GFP protein, and nuclei were visualized with Hoechst 33342 (blue). Supplementing the cell culture medium with the protein was conducted in an equal molar quantity of 5 μM. Scale bar, 50 μm.

A recent study has shown that 30Kc19 protein has a cell-penetrating property when supplemented to the culture medium [32]. In order to examine the ability of 30Kc19 protein to deliver foreign proteins into the cell, a GFP was selected because of its cell-impermeable property and ability to give out its own green fluorescence. This is useful because both intracellular delivery of cell-impermeable cargo is possible and also because of ease of assay via intracellular fluorescence. Thus, GFP-30Kc19 protein expressed from *E. coli*, purified, and was added to culture medium. The results showed that when the 30Kc19 protein is fused with GFP, it was able to penetrate and deliver its cargo protein into cells (Fig. 7). This means that we can utilize the 30Kc19 protein for successful intracellular delivery of cargo proteins.

## 4 Discussion

Cell penetration by the 30Kc19 protein, when added to cell culture medium has only been reported recently [32]. Internalization was shown when 0.2 mg/mL (7 μM) of 30Kc19 protein was added to culture medium (Fig1 and 4A). Other CPP, TAT, was internalized at similar concentration (5 μM) [42]. Under in vitro condition, we noticed the dimerization propensity of 30Kc19 protein (Fig. 2A). We observed that the expressed and purified recombinant 30Kc19 protein, exists as monomer and that dimerization occurs only under SDS and phospholipids. To check the structural changes in the protein in different conditions, various surfactants (cationic, non-ionic, and zwitterionic detergents) were used and tested for dimerization of the 30Kc19 (Fig. 3A). However, dimerization only occurred in the presence of amphiphilic structured moieties (SDS and phospholipids). The results of our analysis showed that the 30Kc19 protein exists both in the form of a monomer (30 kDa) and a dimer (60 kDa) under non-reducing SDS–PAGE conditions. This propensity occurs due to the reducing condition, as the 30Kc19 protein is denatured and all bonds are broken. The protein did not undergo a full denaturation change under fully reduced and non-reduced conditions; hence, formation of the monomer and the dimer. SDS was thought to be involved in the efficient and reversible dimer formation of the 30Kc19 protein, and mixing SDS with the 30Kc19 protein resulted in dimerization when loaded on native PAGE. We found that this propensity was not dependent on the negative charge of the material. However, when phospholipids, which are similar to SDS and a major component of cell membranes, were mixed with the 30Kc19 protein, a 30Kc19 protein dimer formed. The results of adding the cell-penetrating 30Kc19 protein to culture medium showed that the monomer 30Kc19 protein dimerized at the surface of the cell membrane due to the lipid bilayer, penetrated the cells, and remained a dimer inside the cells. The penetrated 30Kc19 protein homodimerized during penetration to cells; hence, the GST pull-down assay was used for further analysis. The result clearly showed that the 30Kc19 monomer protein dimerized with the 30Kc19 protein of the GST-30Kc19 protein during cell penetration. This result indicates that the native 30Kc19 protein exists as a monomer but becomes a homodimer at the surface of the plasma membrane during penetration and remains a homodimer inside cells.

We were convinced that the 30Kc19 protein dimerizes during cell penetration but we were still unsure exactly at which point the 30Kc19 protein homodimerizes during penetration. Mutagenesis of the 30Kc19 protein was conducted to determine at which point the 30Kc19 protein dimerized during penetration. We inferred from another peptide hormone, resistin, that two cysteines in the 30Kc19 protein may play a key role in dimer formation [39, 40]. The results showed that Cys-57 was important for dimerization of the 30Kc19 protein during cell penetration, indicating that dimerization is important for uptake. Figure 5E shows the level of difference in the fluorescence intensity of the protein-treated cells. Fluorescence was clearly weaker for 30Kc19 C76A than that of 30Kc19 protein-treated cells. This result coincided with the PAGE results of Fig. 5C and 5D, in which the 30Kc19 C76A band was weaker than that of the 30Kc19 protein-treated cell lysate. One possible explanation for this outcome could be the lowered protein stability, however, the results showed that all proteins were stable (Fig. 5D). Thus, although Cys-76 was not as important as Cys-57 for cell penetration, it was still quite important. We speculate that Cys-57 forms a dimer with Cys-57 itself and that Cys-57 also forms a dimer with Cys-76. This is why the fluorescence was brighter for 30Kc19 than that for 30Kc19 C76A-treated cells, and showed that more dimerization occurred, hence, more penetration was possible.

Then, intracellular penetration in the presence of inhibitors of endocytosis was demonstrated to verify the method of intracellular entry of the 30Kc19 (Fig. 6). When cells were treated with 30Kc19 protein in a hyperosmolar condition by sucrose, the clathrin-mediated endocytosis was inhibited and no difference in the amount of intracellular 30Kc19 protein was observed. On the other hand, treatment of cytochalasin B or nystatin reduced the cell-penetrating ability of the 30Kc19 protein; demonstrating that 30Kc19 protein penetrates cells by macropinocytosis and caveolin-mediated endocytosis. However, further mechanism study is required to examine which endocytosis pathway is the major penetration route of the 30Kc19 protein.

So far, we have shown the cell-penetrating property of the 30Kc19 protein and the method of its entry to cells by formation of dimer and then by macropinocytosis and/or caveolin-mediated endocytosis. We have then used the cell-penetrating 30Kc19 protein for the intracellular delivery of cargo proteins such as GFP as a fusion partner to examine the ability of 30Kc19 protein to deliver foreign proteins into the cell. As shown in the Fig. 7, when cells were supplemented with GFP-30Kc19 protein it successfully penetrated and intracellularly delivered its cargo, GFP protein. Our results strongly suggest that 30Kc19 protein has great potential for efficient delivery of cell-impermeable cargos for the delivery of micro- and macromolecules including proteins to target intracellularly.

**Figure 8:**
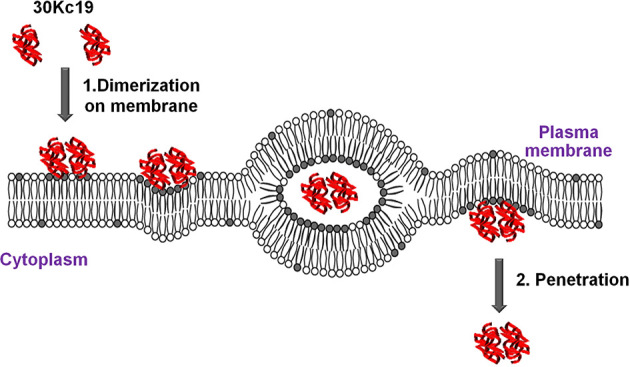
Schematic diagram of 30Kc19 protein monomer that dimerizes at the surface of the cell membrane and penetrates the cell as a dimer. The capability of the 30Kc19 protein to cross the cell membrane efficiently is thought to be similar to other CPP mechanism of entry into cells. The 30Kc19 protein is arbitrarily represented as a dimer in this model. The protein recruits negatively charged phospholipids (black circles) at the plasma membrane. The hydrophilic cavity accommodates the protein and releases it into the cytoplasm.

We observed that the dimerization characteristic of the 30Kc19 protein was similar to a representative protein transduction domain (PTD), Antennapedia (Antp) [34]. This Antp PTD also exhibits a very similar pattern during interactions with SDS and cell membranes [35]. Dimerization of Antp also has the characteristic of penetrating inside cell in an energy independent manner. In addition, dimerization propensities of the fibroblast growth factor receptors 3 (FGFR3) transmembrane domains in detergents and in lipid bilayers were observed previously [43]. Like the 30Kc19 protein, cysteine was involved in the propensity for dimerization, which suggests that the nature of the hydrophobic environment plays an important role in defining the structure and characteristic of proteins. The propensity of the 30Kc19 protein to dimerize is thought to be similar manner to TM domain of FGFR3, and the cell penetrating mechanism is thought to be similar to Antp mechanism of entry into cells (Fig. 8). Through this finding, we anticipate the use of the 30Kc19 protein for the efficient protein delivery system and also in the finding of a novel cell-penetrating peptide around Cys-57 for efficient delivery of cargo inside various cells. The 30Kc19 protein is a non-virus derived (e.g. TAT) CPP, thus intracellular cargo delivery using the 30Kc19 protein may open up new approaches for the delivery of therapeutics in bioindustries, such as pharma- and cosmeceuticals.

## Abbreviations:

Antp: Antennapedia
CPP: cell-penetrating protein/peptide
CTAB: cetyl trimethylammonium bromide
GST: glutathione-*S*-transferase
SDS: sodium dodecyl sulfate
